# Extra-Nuclear Signaling Pathway Involved in Progesterone-Induced Up-Regulations of p21^cip1^ and p27^kip1^ in Male Rat Aortic Smooth Muscle Cells

**DOI:** 10.1371/journal.pone.0125903

**Published:** 2015-05-01

**Authors:** Hui-Chen Wang, Sung-Po Hsu, Wen-Sen Lee

**Affiliations:** 1 Graduate Institute of Medical Sciences, College of Medicine, Taipei Medical University, Taipei 110, Taiwan; 2 Department of Physiology, School of Medicine, College of Medicine, Taipei Medical University, Taipei 110, Taiwan; 3 Cancer Research Center, Taipei Medical University Hospital, Taipei 110, Taiwan; National Cheng Kung University, TAIWAN

## Abstract

Previously, we demonstrated that progesterone (P4) at physiologic levels (5-500 nM) inhibited proliferation in cultured rat aortic smooth muscle cells (RASMCs) through a P4 receptor (PR)-dependent pathway. We also showed that P4-induced cell cycle arrest in RASMCs occurs when the cyclin-CDK2 system is inhibited just as p21^cip1^ and p27^kip1^ protein levels are augmented. In the present study, we further investigated the molecular mechanism underlying P4-induced up-regulations of p21^cip1^ and p27^kip1^ in RASMCs. We used pharmacological inhibitors as well as dominant negative constructs and conducted Western blot analyses to delineate the signaling pathway involved. Our data suggest that P4 up-regulated the expression of p21^cip1^ and p27^kip1^ in RASMCs through increasing the level of p53 protein mediated by activating the cSrc/Kras/Raf-1/AKT/ERK/p38/IκBα/NFκB pathway. The findings of the present study highlight the molecular mechanism underlying P4-induced up-regulations in p21^cip1^ and p27^kip1^ in RASMCs.

## Introduction

Atherogenesis, a degenerative process involving a variety of lesions of the arterial wall, is a response of vascular endothelial cells and smooth muscle cells to injury [1–4]. Vascular smooth muscle cells reside in the media of blood vessels and compose the majority of the vessel wall. Normally, vascular smooth muscle cells have a very low proliferative activity. However, in response to injury or to various stimuli, vascular endothelial cells are activated and produce cytokines as well as growth factors to promote proliferation and migration of vascular smooth muscle cells [3]. Damage to the endothelium might also induce inflammatory responses involved in the development of atherosclerosis. Generally, atherosclerosis is initiated by inflammatory processes of the endothelium that retain low-density lipoprotein particles and circulating monocytes. The attached monocytes then migrate to the subendothelial space, where they are further activated to become monocyte-derived macrophages. In the subendothelial space, these macrophages ingest oxidized low-density lipoprotein and then turn into large foam cells contributing to the formation of the necrotic core and thinning of the fibrous cap [5].

Epidemiological studies indicated that morbidity and mortality from almost all forms of vascular disease in pre-menopausal women are much lower than in age-matched men. The incidence of cardiovascular disease in post-menopausal women gradually approaches that in age-matched men [6], and estrogen replacement reduces the incidence of cardiovascular diseases in the postmenopausal women [7]. These findings suggest that endogenous female sex hormones might have a protective effect against atherosclerotic vascular diseases during pre-menopausal years. This hypothesis is supported by the evidence that estradiol (E2) administration inhibits the development of atherosclerosis in experimental animals [8–13].

While the effects of E2 on cardiovascular diseases have been intensively studied, little is known about the effect of progesterone (P4). Previously, Grodstein et al. showed that the relative risk of major coronary heart disease among post-menopausal women who took E2 with P4 together is lower compared to the risk of those who took E2 alone [14]. Moreover, castrated baboons receiving E2 and P4 together had fewer vascular lesions than those receiving E2 alone [15]. Miyagawa et al. also showed that combined treatment with P4 and E2 could protect coronary vasospasm from pathophysiological stimulation in ovariectomized rhesus monkeys [16]. However, some other reports indicated that the combined E2 and P4 trial did not confirm a protective effect against cardiovascular diseases [17, 18]. Although the effect of P4 on cardiovascular diseases has become an interesting issue, there is little evidence for the effect of P4 alone on atherosclerosis.

Previously, we demonstrated that physiologic levels of P4, but not E2, inhibit DNA synthesis and decrease cell number in cultured rat and human aortic smooth muscle cells in a concentration-dependent manner [19]. Our data indicate that P4-induced cell cycle arrest in RASMCs occurs when the cyclin-CDK system is inhibited just as p21^cip1^ and p27^kip1^ protein levels are augmented [20]. We also demonstrated that P4 inhibits the migration of RASMCs through inhibiting the RhoA activity mediated by up-regulation of p27^kip1^ expression [21–23]. Taken together, these data suggest that P4 alone could inhibit the vascular smooth muscle cell proliferation and migration. Here, we further investigate the molecular mechanism underlying P4-induced up-regulations of p21^cip1^ and p27^kip1^ in RASMCs. The findings of this study provide important insights into the molecular and cellular mechanisms of atheroprotective effects of P4. Only when the mechanism of atheroprotective effects of P4 is fully understood can we begin to design a strategy for preventing and treating atherosclerosis and its complications.

## Materials and Methods

### Cell cultures

Rat aortic smooth muscle cells (RASMCs) were harvested from the thoracic aortas of adult male Wistar rats (250–300 g) by enzymatic dissociation. This study was carried out in strict accordance with the recommendations in the Guide for the Care and Use of Laboratory Animals of the Taiwan National Institutes of Health. The protocol was approved by the Committee on the Ethics of Animal Experiments of the Taipei Medical University (Permit Number: LAC-2013-0196). All surgeries were performed under sodium pentobarbital anesthesia, and all efforts were made to minimize suffering. After vascular smooth muscle cell isolation, the animal was sacrificed using CO_2_. The isolated RASMCs were grown in DMEM (GIBCO, Grand Island, NY) supplemented with 10% fetal bovine serum (FBS; GIBCO), penicillin (100 U/mL), and streptomycin (100 μg/mL, GIBCO) in a humidified 37°C and 5% CO_2_ incubator. Cells from passages 5–10 were used. For the experiments, RASMCs were treated with either P4 (50 nM) in 0.05% DMSO (Sigma-Aldrich, St. Louis, MO) for the indicated times or 0.05% DMSO (control).

### Reagents

Antibodies against p-AKT, AKT, cadherin, p-ERK, p21^cip1^, p-p38, p38, p-Raf-1, PARP, p65, p-IκBα, or IκBα were purchased from Santa Cruz Biotechnology, Inc. (Santa Cruz, CA). Anti-α-tubulin, anti-ERK, and anti-p27^kip1^ antibody were purchased from Sigma-Aldrich. Anti-G3PDH antibody was purchased from GeneTex (Hsinchu, Taiwan). Anti-cSrc antibody was purchased from Abcam (Cambridge, MA). Anti-p-cSrc antibody was purchased from Cell Signaling Technology Inc. (Beverly, MA). Anti-Raf-1 antibody was purchased from Epitomics (Burlingame, CA). PP2 was purchased from A. G. Scientific, Inc. (San Diego, CA). SB 203580 and Bay 11–7082 were purchased from Cayman Chemical (Ann Arbor, MI).

### Western blot analysis

To determine the protein levels in RASMCs, total proteins were extracted and Western blot analyses were performed as described previously [20]. Electrophoresis was carried out using a 12% SDS-polyacrylamide gel (50 μg protein per lane). Separated proteins were transferred onto PVDF membranes, treated with 1% BSA/0.02% NaNO_3_ to block the non-specific IgGs, incubated for 1 h with specific primary antibody (0.2 μg/mL), and then incubated with second antibody (Jackson ImmunoResearch Laboratories, West Grove, PA) linked to horse radish peroxidase (1:10,000) for 1 h. Subsequently, the blot was developed using the ECL (enhanced chemiluminescence) system (GE, Healthcare, NJ). The intensity of each band was quantified by densitometry analysis using Image Pro-Plus 4.5 Software.

### Subcellular fractionation

To examine the membrane translocation of Kras, RASMCs were washed with ice-cold PBS and re-suspended in hypotonic buffer (20 mM Tris-HCl, pH 7.5, 5 mM EGTA, 2 mM EDTA, 1 mM NaVO_3_, 1 mM DTT, 10 mM NaF, 10 mM Na_2_H_2_P_2_O_7_) plus protease inhibitor cocktail (Sigma-Aldrich). After being frozen at -80°C for 2 h, the cell lysates were disrupted by passing through a 3/10 mL BD insulin syringe and 30-gauge needle for 120 times, and then incubated on ice with repeated vortex mixing for 6 times (5 min each time). The supernatant was collected as the cytosolic fraction after being centrifuged at 14,000 g for 10 min at 4°C. Pellets were washed with ice-cold PBS for 2 times, and then lysed in the extract buffer (50 mM HEPES, pH 7.0, 250 mM NaCl, 2.5 mM EDTA, 1% NP-40, 5% Glycerol, 1 mM NaVO_3_, 10 mM NaF, 10 mM Na_2_H_2_P_2_O_7_) plus protease inhibitor cocktail (Sigma-Aldrich) on ice with repeated vortex mixing for 9 times (5 min in each), and then centrifuged at 14,000 g for 30 min at 4°C. The supernatant was collected as the particulate (membrane) fraction.

### Nuclear extraction

To examine the effect of P4 on nuclear translocation of NFκB (p65), the NE-PER nuclear and cytoplasmic extraction reagents (Thermo Fisher Scientific, Rockford, IL) was used and the extraction was performed as previously described [24].

### Cell transfection

For transient transfection of the indicated constructs into RASMCs, jetPEI-RASMCs transfection reagent (Polyplus Transfection, Bioparc, France) was used and the transfection was performed as previously described [21, 25].

### Statistical analysis

All data were expressed as the mean value ± s.e.mean. Comparisons were subjected to one way analysis of variance (ANOVA) followed by Fisher’s least significant difference test. Significance was accepted at *P* < .05.

## Results

### Candidate molecules involved in the P4-induced proliferation inhibition in RASMCs

**Fig 1:**
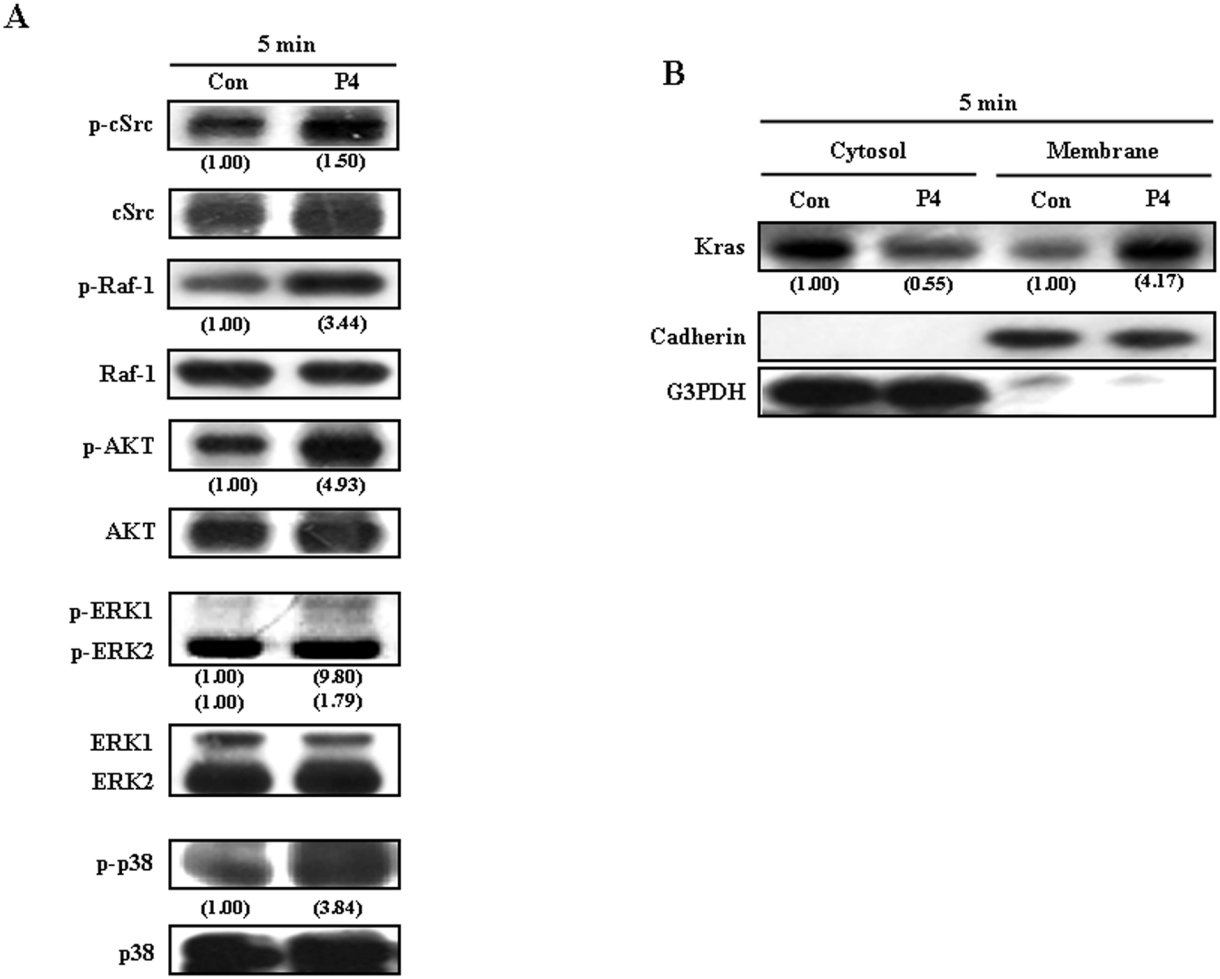
Activations of candidate proteins involved in P4-induced increases in the levels of p21^cip1^ and p27^kip1^ protein in RASMCs. Treatment of RASMCs with P4 (50 nM) for 5 min increased the levels of p-cSrc, p-Raf-1, p-AKT, p-ERK1/2, and p-p38 protein (A) and membrane translocation of Kras, an indicator for Kras activation (B). Data are representative of 2 independent experiments with similar results. Values shown in parentheses represent the quantified results adjusted with their own total protein level (A) or with G3PDH (for cytosol protein) and cadherin (for membrane protein), and expressed as ratio over control. Con, control.

To delineate the signaling pathway involved in the P4-induced up-regulations of p21^cip1^ and p27^kip1^ in RASMCs, we initially examined the activity changes of several candidate molecules, which have been indicated to be involved in regulating cell proliferation, in P4-treated RASMCs. Since cSrc, Kras, Raf, AKT, ERK, and p38 [26–29] have been suggested to play important roles in regulating cell proliferation, we examined the effect of P4 treatment on the activity of these candidate molecules. Our results showed that treatment with P4 (50 nM) for 5 min increased the levels of p-cSrc, p-Raf-1, p-ERK1/2, p-AKT, and p-p38 protein (Fig 1A) and membrane translocation of Kras (Fig 1B) in RASMCs.

### Involvement of cSrc activation in P4-induced increases in p21^cip1^ and p27^kip1^ protein in RASMCs

**Fig 2:**
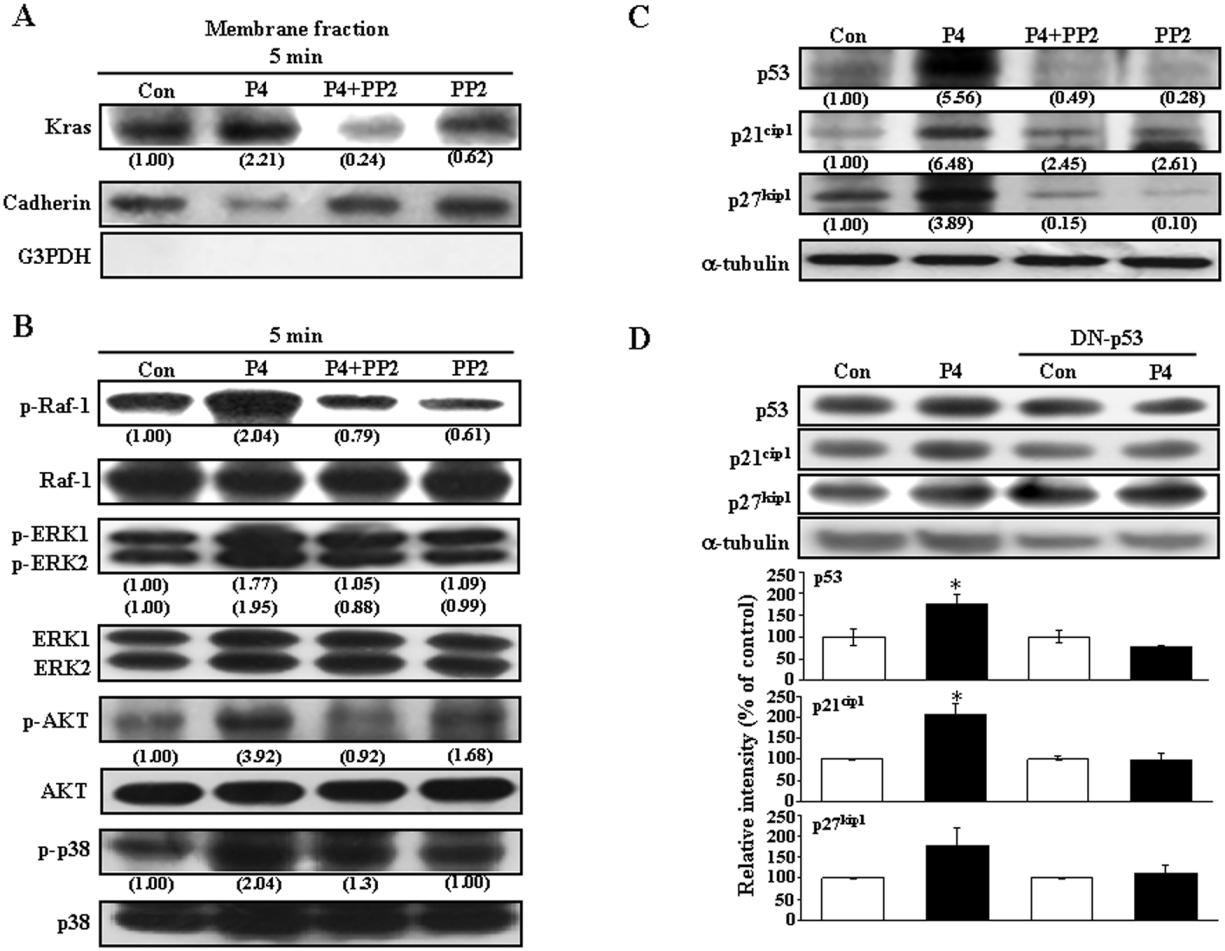
cSrc is the most upstream molecule involved in P4-induced increases in the levels of p21^cip1^ and p27^kip1^. Pre-treatment with PP2 (200 nM) for 1 h prevented P4-induced membrane translocation of Kras (A), increases in the levels of p-Raf-1, p-ERK1/2, p-AKT and p-p38 protein (B), and p21^cip1^, p27^kip1^ and p53 protein (C). Data are representative of 2 independent experiments with similar results. Values shown in parentheses represent the quantified results adjusted with G3PDH and cadherin for cytosol and membrane, respectively (A), with their own total protein level (B), or with α-tubulin (C) and expressed as ratio over control. Con, control.

Previously, we demonstrated that PR and cSrc forms a complex in RASMCs and the level of p-cSrc-PR complex is increased after 10 sec treatment with P4 [21], suggesting that cSrc is the most upstream molecule involved in P4-regulated cell behaviors. Since cSrc has been implicated in the regulation of cell proliferation [26], we studied whether activation of cSrc was involved in the P4-induced up-regulations of p21^cip1^ and p27^kip1^ in these RASMCs. Pre-treatment with a cSrc inhibitor, PP2 (200 nM), abolished the P4 (50 nM)-induced membrane translocation of Kras (Fig 2A) and increases in the levels of p-Raf-1, p-ERK1/2, p-AKT, and p-p38 protein (Fig 2B). P4-induced increases in the levels of p21^cip1^, p27^kip1^ and p53 protein (Fig 2C) were also prevented by pre-treatment with PP2. These findings suggest that cSrc is the most upstream molecule involved in regulating the P4-induced up-regulations of p21^cip1^ and p27^kip1^.

### The cSrc-mediated signaling pathway involved in P4-induced up-regulations of p21^cip1^ and p27^kip1^ protein in RASMCs

**Fig 3:**
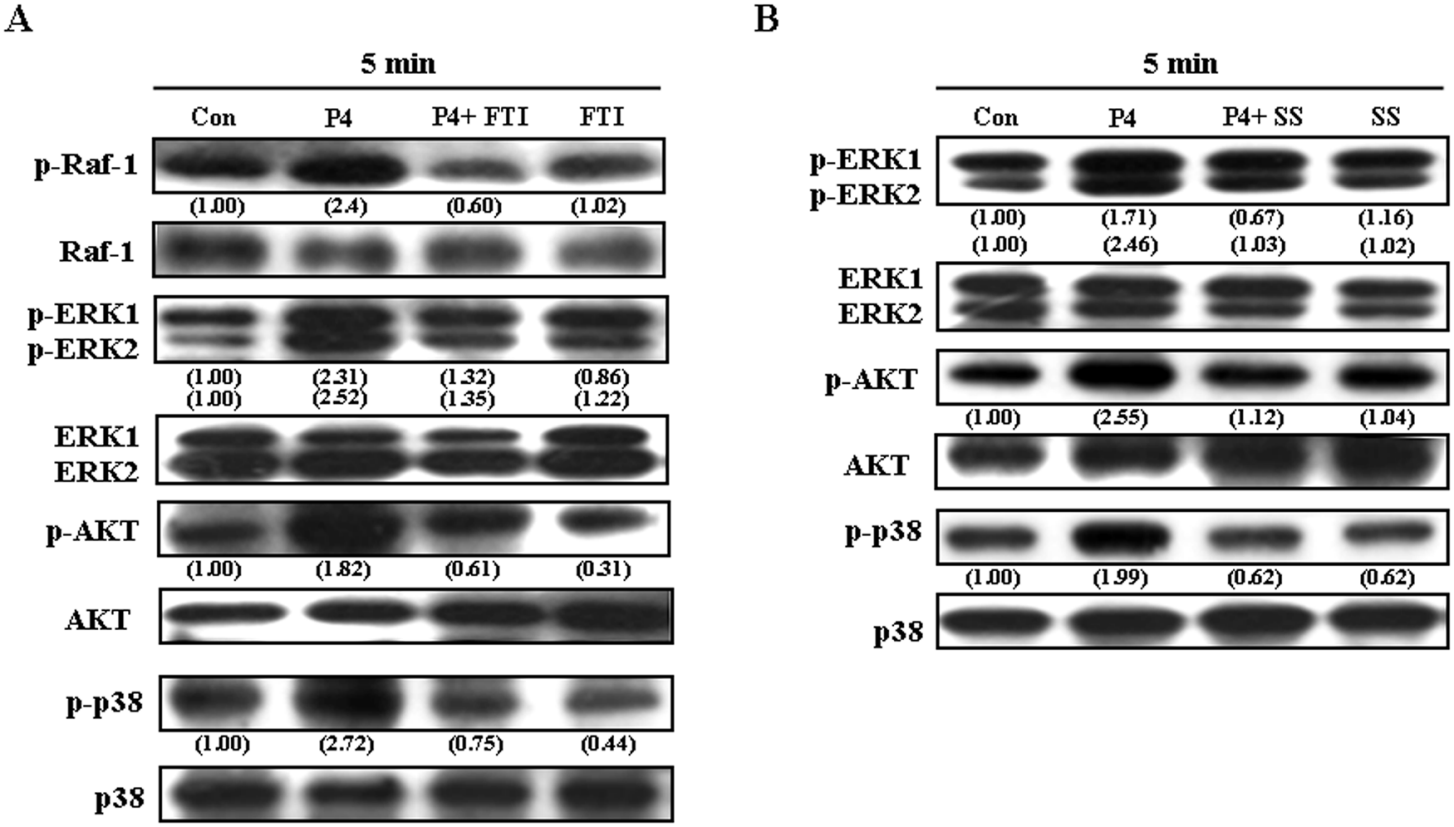
Kras and Raf-1 are the upstream molecules of the AKT/ERK1/2/p38 pathway involved in P4-induced increases in the levels of p21^cip1^ and p27^kip1^ protein in RASMCs. (A) Pre-treatment of RASMCs with a Kras inhibitor, FTI (10 μM), for 1 h prevented P4-induced increases in the levels of p-Raf-1, p-ERK1/2, p-AKT, and p-p38 protein. (B) Pre-treatment of RASMCs with a Raf-1 inhibitor, sulindac sulfide (10 μM), for 1 h prevented P4-induced increases in the levels of p-ERK1/2, p-AKT, and p-p38 protein. Data are representative of 2 independent experiments with similar results. Values shown in parentheses represent the quantified results adjusted with their own total protein level and expressed as ratio over control. Con, control. FTI, farnesyltransferase inhibitor; SS, sulindac sulfide.

**Fig 4:**
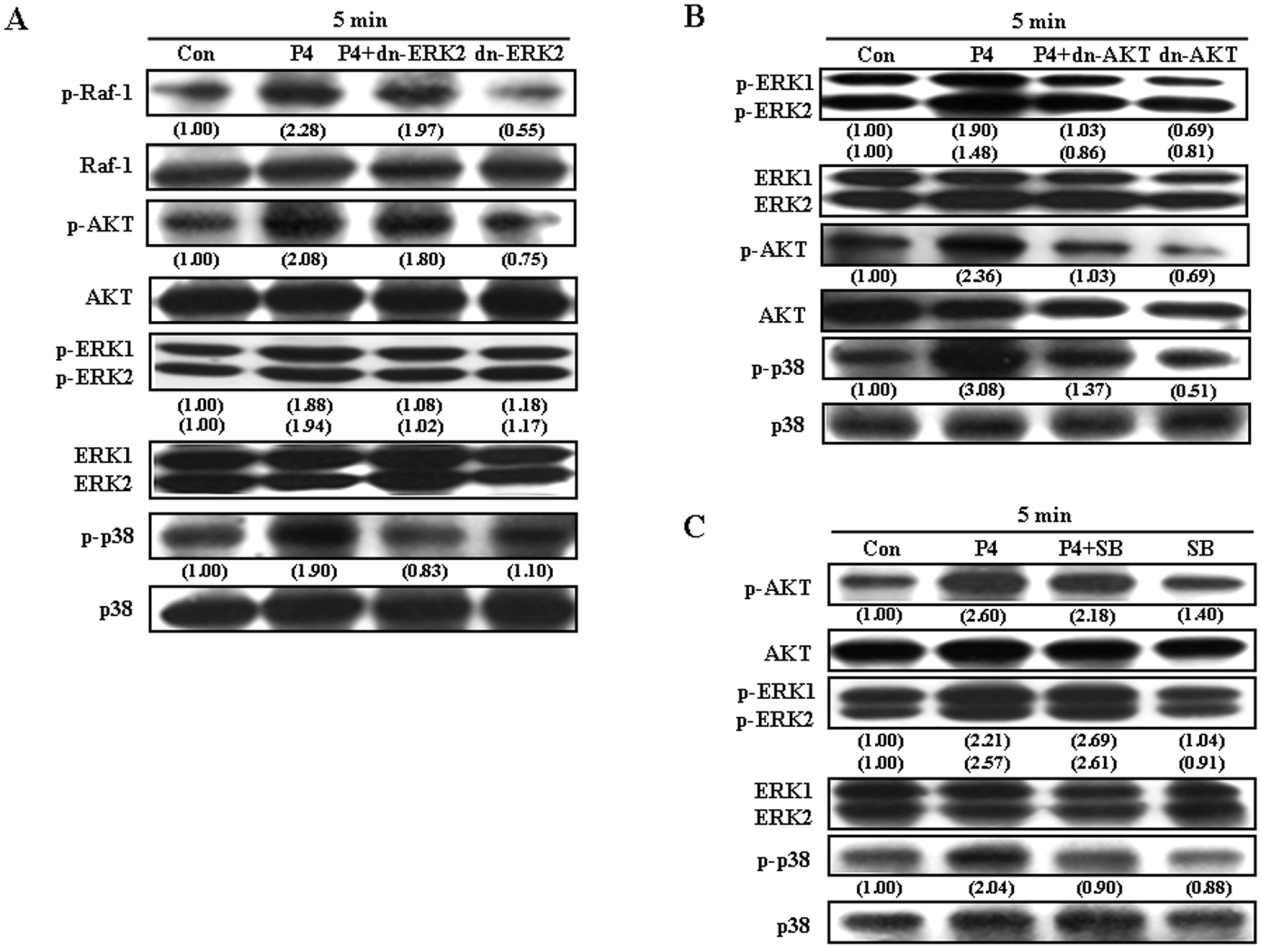
Involvement of AKT/ERK/p38 in P4-induced increases in the levels of p21^cip1^ and p27^kip1^ protein in RASMCs. (A) Pre-transfection of RASMCs with dn-ERK2 cDNA prevented P4-induced increases in the levels of p-ERK1/2, and p-p38 protein, but not p-Raf-1 and p-AKT protein. (B) Pre-transfection of RASMCs with dn-AKT cDNA prevented P4-induced increases in the levels of p-AKT, pERK1/2, and p-p38 protein. (C) Pre-treatment of RASMCs with a p38 inhibitor, SB 203580 (1 μM), for 1 h prevented P4-induced increases in the level of p-p38 protein, but not p-AKT and p-ERK1/2 protein. Data are representative of 2 independent experiments with similar results. Values shown in parentheses represent the quantified results adjusted with their own total protein level and expressed as ratio over control. Con, control; dn, dominate negative; SB, SB 203580.

We next examined the cSrc downstream molecules involved in P4-induced up-regulations of p21^cip1^ and p27^kip1^ protein in RASMCs. As shown in Fig 3A, pre-treatment with a Ras inhibitor, farnesyltransferase inhibitor (FTI, 10 μM), abolished P4-induced increases in Raf-1, ERK1/2, AKT, and p38. Raf-1 is a direct effector protein of Kras. Pre-treatment with a Raf-1 inhibitor, sulindac sulfide (10 μM), abolished P4-induced increases in p-ERK1/2, p-AKT, and p-p38 protein (Fig 3B), suggesting that Raf-1 molecule is downstream of Kras and upstream of ERK1/2, AKT and p38. Fig 4A demonstrated that pre-transfection with dominant negative ERK 2 (dn-ERK 2) cDNA, abolished P4-induced increases in p-ERK1/2 and p-p38, but not p-Raf-1 and p-AKT in RASMCs. On the other hand, pre-transfection with dn-AKT cDNA abolished P4-induced increases in the levels of p-AKT, p-ERK1/2 and p-p38 protein (Fig 4B), suggesting that ERK1/2 is downstream of AKT and upstream of p38. To confirm this conclusion, RASMCs were treated with a p38 inhibitor, SB 203580 (1 μM), prior to P4 treatment. As shown in Fig 4C, SB 203582 abolished P4-induced increases in the level of p-p38 protein, but did not affect the levels of p-AKT protein and p-ERK1/2, suggesting that p38 is downstream of AKT and ERK1/2.

**Fig 5:**
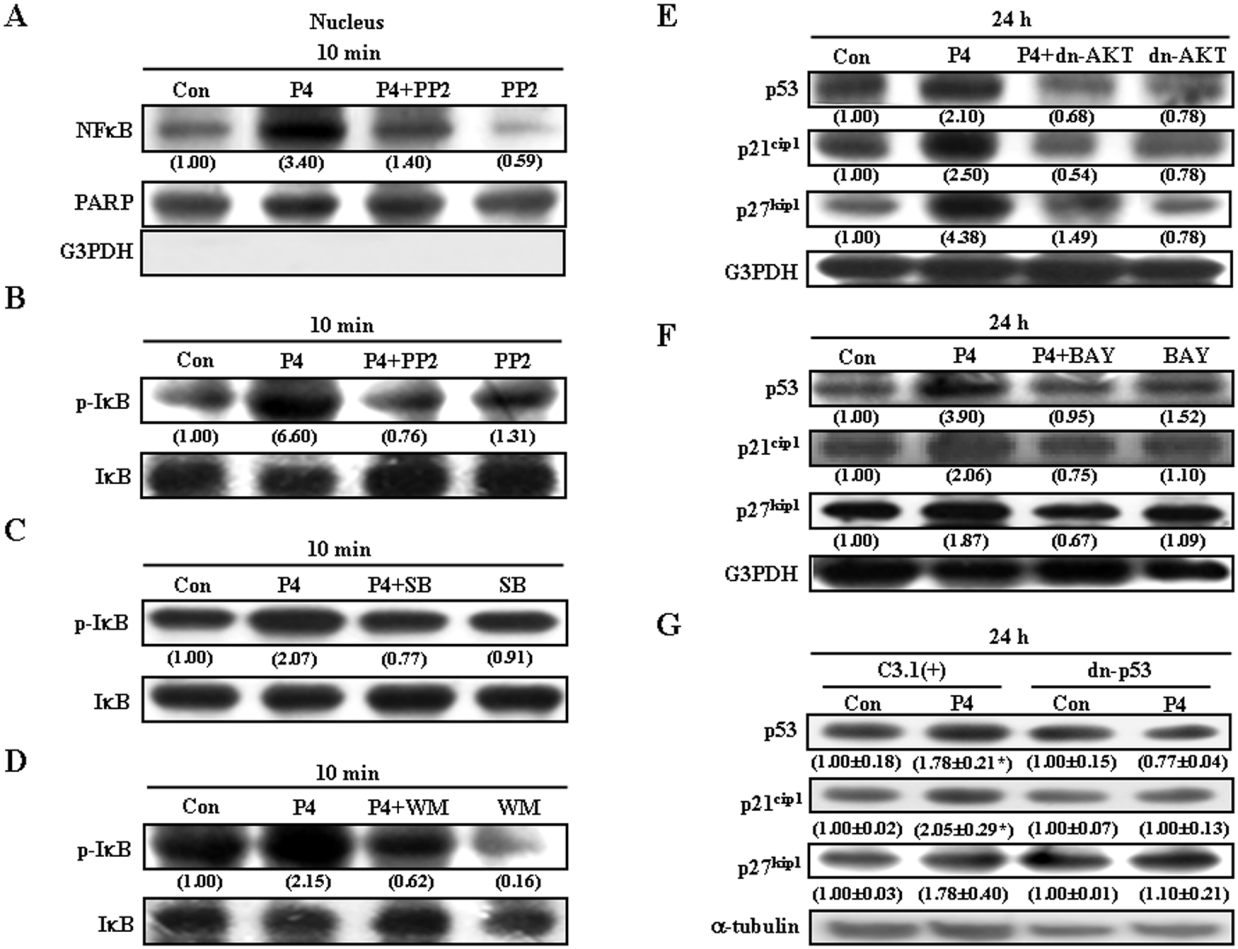
The cSrc/Kras/Raf-1/AKT/ERK/p38-mediated pathway is involved in P4-induced NFκB (p65) nuclear translocation. (A) Pre-treatment of RASMCs with PP2 (200 nM) for 1 h prevented the P4-induced NFκB nuclear translocation. Pre-treatment of RASMCs with 200 nM PP2 (B), 1 μM SB 203580 (C), or 100 nM wortmannin (D) prevented P4-induced increases in the levels of p-IκBα. (E) Pre-transfection with dn-AKT cDNA prevented P4-induced increases the levels of p53, p21^cip1^ and p27^kip1^ protein. (F) Pre-treatment with BAY (10 nM) prevented P4-induced increases in the levels of p53, p21^cip1^ and p27^kip1^ protein. (A-F) Data are representative of 2 independent experiments with similar results. Values shown in parentheses represent the quantified results adjusted with their own total protein level, RARP (for nuclear protein) or G3PDH (for cytosolic protein) and expressed as ratio over control. (G) Pre-transfection with dn-p53 cDNA prevented P4-induced increases in the levels of p21^cip1^and p27^kip1^ protein. Values shown in parentheses represent the quantified results adjusted with α-tubulin protein level and expressed as ratio over control. Values represent the means±s.e.mean. (n = 3). *P < 0.05 different from control group. BAY, BAY11-7082; Con, control; C3.1 (+), pcDNA 3.1 (+); dn, dominate negative; SB, SB 203580; WM, wortmannin.

We previously demonstrated that P4 increases p21^cip1^ and p27^kip1^ expression through the NFκB (p65)-induced up-regulation of p53 in HUVEC [24]. Accordingly, we further examined the involvement of NFκB activation in P4-induced up-regulations of p21^cip1^ and p27^kip1^ in RASMCs. As illustrated in Fig 5A, P4 (50 nM) increased nuclear translocation of p65 protein, and this effect was abolished by pre-treatment of the cell with PP2. Since phosphorylation, ubiquitination, and degradation of the IκBα protein have been demonstrated to result in dissociation of NFκB from IκBα, and thereby allow NFκB to migrate into the nucleus and activate gene expression [30, 31], we further examined the effect of P4 treatment on phosphorylation of IκBα. P4 (50 nM) induced an increase in the level of p-IκBα, and this effect was abolished by pre-treatment of the cell with PP2 (Fig 5B), SB 203580 (Fig 5C), or a PI3K inhibitor, Wortmannin (100 nM) (Fig 5D). To further confirm that activation of the cSrc/Kras/Raf-1/AKT/ERK1/2/p38/IκBα/NFκB signaling pathway is involved in P4-induced increases in p21^cip1^ and p27^kip1^ in RASMCs, the effect of inhibition of AKT activation and NFκB nuclear translocation was examined. Pre-transfection with dn-AKT (Fig 5E) or pre-treatment with an NFκB inhibitor, BAY11-7082 (Fig 5F), prevented P4-induced increases in the levels of p53, p21^cip1^ and p27^kip1^ protein.

### Involvement of p53 in P4-induced up-regulations of p21^cip1^ and p27^kip1^ in RASMCs

To investigate whether P4-induced up-regulation of p53 protein participates in up-regulations of p21^cip1^ and p27^kip1^ protein in RASMCs, RASMCs were transfected with dn-p53 cDNA. As shown in Fig 5G, pre-transfection of RASMCs with dn-p53 cDNA prevented P4-induced increases in the levels of p21^cip1^ and p27^kip1^ protein, suggesting that P4-induced increases of p21^cip1^ and p27^kip1^ protein were regulated through up-regulating the p53 protein.

## Discussion

Previously, we demonstrated that P4 at physiologic levels (5–500 nM) caused cell cycle arrest in RASMCs through up-regulations of p21^cip1^ and p27^kip1^ protein [19, 20]. In the present study, we further delineated the molecular mechanism underlying P4-induced up-regulations of p21^cip1^ and p27^kip1^ protein in RASMCs. The cSrc/Kras/Raf-1/AKT/ERK1/2/p38/IκB/NFκB/p53 signaling pathway in RASMCs is activated by P4. Blockade of this pathway abolished P4-induced up-regulations of p21^cip1^ and p27^kip1^. To our knowledge, this is the first demonstration that extra-nuclear activation of the cSrc/Kras/Raf-1/AKT/ERK1/2/p38/IκB/NFκB/p53 signaling pathway is involved in the P4-induced up-regulations of p21^cip1^ and p27^kip1^ expression in RASMCs.

The PR is a ligand-activated steroid receptor member of the nuclear receptor superfamily. Traditionally, P4 is thought to mediate its biological effects through binding to the PR, restructuring with dimerization, entering the nucleus, and binding to DNA to alter gene transcription. Lately, a growing body of evidence has suggested that PR has dual functions as a nuclear transcription factor and a modulator of cell signaling pathway. In the cytosol, P4 binds to PR, forms dimers, and then translocates to the nucleus, where the P4/PR complex binds to the promoter and acts as a transcription factor to regulate gene expressions. In contrast, the putative membrane-associated PR activated by P4 activates signaling pathways and induces rapid effects independently of transcriptional or genomic actions [32]. Previously, we showed that PR and cSrc forms a complex in RASMCs. Moreover, the level of p-Src-PR complex in RASMCs was increased at 10 sec after P4 treatment and this effect was abolished by pre-treatment of the cell with a PR antagonist, suggesting that P4-induced activation of cSrc is mediated through a non-genomic action of P4 [21]. This rapid action of P4 in the cytosol and consequent activation of cSrc has also been implicated in the regulation of cell cycle and migration [33]. Further indication that cSrc activation is involved in P4-induced up-regulations of p21^cip1^, p27^kip1^ and p53 was evidenced by failure of these effects of P4 to occur in PP2-treated RASMCs (Fig 2C). Taken together, these results suggest that activation of the cSrc-mediated signaling pathway might be involved in the P4-induced up-regulations of p21^cip1^, p27^kip1^ and p53 in RASMCs.

It has been reported that P4 can activate the cSrc/Ras/MAP kinase signaling pathway in T47D cells [34]. In the present study, we showed that P4 treatment increased the membrane translocation of Kras (Fig 1B) and levels of p-Raf-1, p-ERK1/2, p-AKT, and p-p38 (Fig 1A). Pretreatment of RASMCs with a Src kinase inhibitor, PP2, blocked P4-induced increases in membrane translocation of Kras (Fig 2A), levels of p-Raf-1, p-ERK1/2, p-AKT, p-p38, and p-IκBα protein (Fig 2B), nuclear translocation of NFκB (Fig 5A), and levels of p21^cip1^, p27^kip1^ and p53 protein (Fig 2C). Using immunoprecipitation and chromatin-immunoprecipitation analyses, we recently showed that P4 increases both the formation of PR-NFκB complex in the nucleus and the binding of PR onto the NFκB binding fragment of the p53 promoter in HUVEC. Ablation of the NFκB binding motif in the p53 promoter completely abolishes P4-induced increases in the p53 promoter activity. These results suggest that the PR-NFκB complex formation is required for mediating p53 expression [35]. Taken together, these findings further suggest that P4-induced up-regulations of p21^cip1^ and p27^kip1^ were mediated through a non-genomic action of PR.

It has been indicated that activation of Kras can stimulate the PI3K/AKT pathway, which plays an important role in cell survival and proliferation [36]. A previous report showed that AKT can associate with p21^cip1^ and phosphorylates p21^cip1^, resulting in cytoplasmic localization of p21^cip1^, which in turn increases cell proliferation [37]. A similar effect of AKT on p27^kip1^ is also reported [38, 39]; AKT induces phosphorylation of p27^kip1^, which in turn causes retention of p27^kip1^ in the cytoplasm, precluding p27^kip1^-induced cell cycle arrest. Surprisingly, our previous data indicate that P4-induced cell cycle arrest in RASMCs is mediated through increasing the expression of p21^cip1^ and p27^kip1^ and our present data suggest that P4-induced up-regulations of p21^cip1^ and p27^kip1^ in RASMCs were mediated through activation of AKT. The regulation of cell proliferation in multicellular organism is a complex process regulated primarily by external growth factors. The signaling pathway involved in the stimulus-regulated cell proliferation depends on the cell type and stimulus. The MAP kinase-mediated signaling pathways represent several families of signal transduction cascades that mediate information provided by extracellular stimuli. Although it has been indicated that Raf/AKT/ERK activation is important in cell proliferation, the role of Raf/AKT/ERK pathway in regulating cell cycle progress depends on the cell type and stimulus. We have recently demonstrated that Ras/Raf/ERK activation might also contribute to cell cycle arrest in magnolol-treated COLO-205 [40], P4-treated HUVEC [24], and folic acid-treated HUVEC [41]. In HUVEC, we previously demonstrated that P4 binds to PR, subsequently activating the cSrc/Kras/Raf-1/ERK2 signaling cascade to increase NFκB nucleus translocation and binding onto the p53 promoter, which in turn up-regulates the expression of p53, eventually resulting in the inhibition of endothelial cell proliferation. The data from the present study suggest that P4 inhibited RASMC proliferation through a similar signaling pathway found in the P4-induced proliferation inhibition in HUVEC.

P21^cip1^ and p27^kip1^ are two known cyclin-dependent kinase-inhibitory proteins. P21^cip1^ is a transcriptional target of the tumor suppressor protein p53 [42, 43]. Expression of p21^cip1^ is tightly controlled by wild-type but not mutant p53 [42]. Several studies have shown that expression of p53 in the cells can induce growth arrest through transcriptional activation of p21^cip1^ [24, 44, 45]. P27^kip1^ is also thought to play an important role in negative regulation of cell division and to mediate growth arrest in vivo [46]. Targeted disruption of the mouse p27^kip1^ gene has been demonstrated to result in enhanced growth of mice, multiple organ hyperplasia, and a predisposition to tumors [47–49]. It has been demonstrated that the Ras-mediated pathway might regulate p27^kip1^ expression [50]. However, there is little evidence on the p53-mediated regulation of p27^kip1^ expression. Previously, we have demonstrated that both folic acid and P4 can induce increases in p21^cip1^ and p27^kip1^ in human endothelial cells through up-regulation of p53 [41]. The evidences for regulation of p27^kip1^ expression by p53 protein from our previous studies include that (1) pre-transfection with dn-p53 cDNA abolishes P4-induced increases in p27^kip1^ promoter activity and protein level; (2) pre-transfection with p53 siRNA abolishes P4-induced increases in p27^kip1^ protein level; (3) the p53 DNA binding activity on the p27^kip1^ promoter is activated by P4. In this study, we showed that P4 increased the levels of p53 protein in RASMCs. Transfection with dn-p53 cDNA abolished P4-induced increases in the levels of p21^cip1^ and p27^kip1^ protein, suggesting that P4-induced up-regulations in p21^cip1^ and p27^kip1^ in RASMCs through a p53-dependent manner. This finding further support the regulation of p27^kip1^ expression by p53 protein.

The effect of p53 on the p27^kip1^ expression is through a direct or an indirect regulation is an important issue. FoxO3, a potent transcriptional activator, has been indicated to play a crucial role in provoking expression of a cluster of genes in controlling DNA repair, apoptosis and cell cycle arrest. FoxO3 has been shown to be a direct downstream transcriptional target of p53 in both mouse embryonic fibroblasts and in thymocytes [51] and to be capable of inhibiting cell cycle progression via a direct up-regulation of p27^Kip1^ [52]. Therefore, the assumption that FoxO3 might mediate the p53-induced up-regulation of p27 ^Kip1^ has been proposed. However, the report by Renault et al. suggested that FoxO3 may not be necessary for p53-dependent cell cycle arrest, but appears to modulate p53-dependent apoptosis [51]. In addition, our recent studies showed that the promoter -257 ~ -309 fragment of p27 ^Kip1^ containing three p53 binding domains is required for p27Kip1 promoter activity in P4-treated HUVEC. Although these findings suggest that FoxO3 may not be involved in p53-induced up-regulation of p27 ^Kip1^ under the stimulation of DNA damage agents and P4, the possible linkage of p53-FoxO3-p27 ^Kip1^ axis in triggering cell cycle arrest still can not be ruled out under different circumstances.

**Fig 6:**
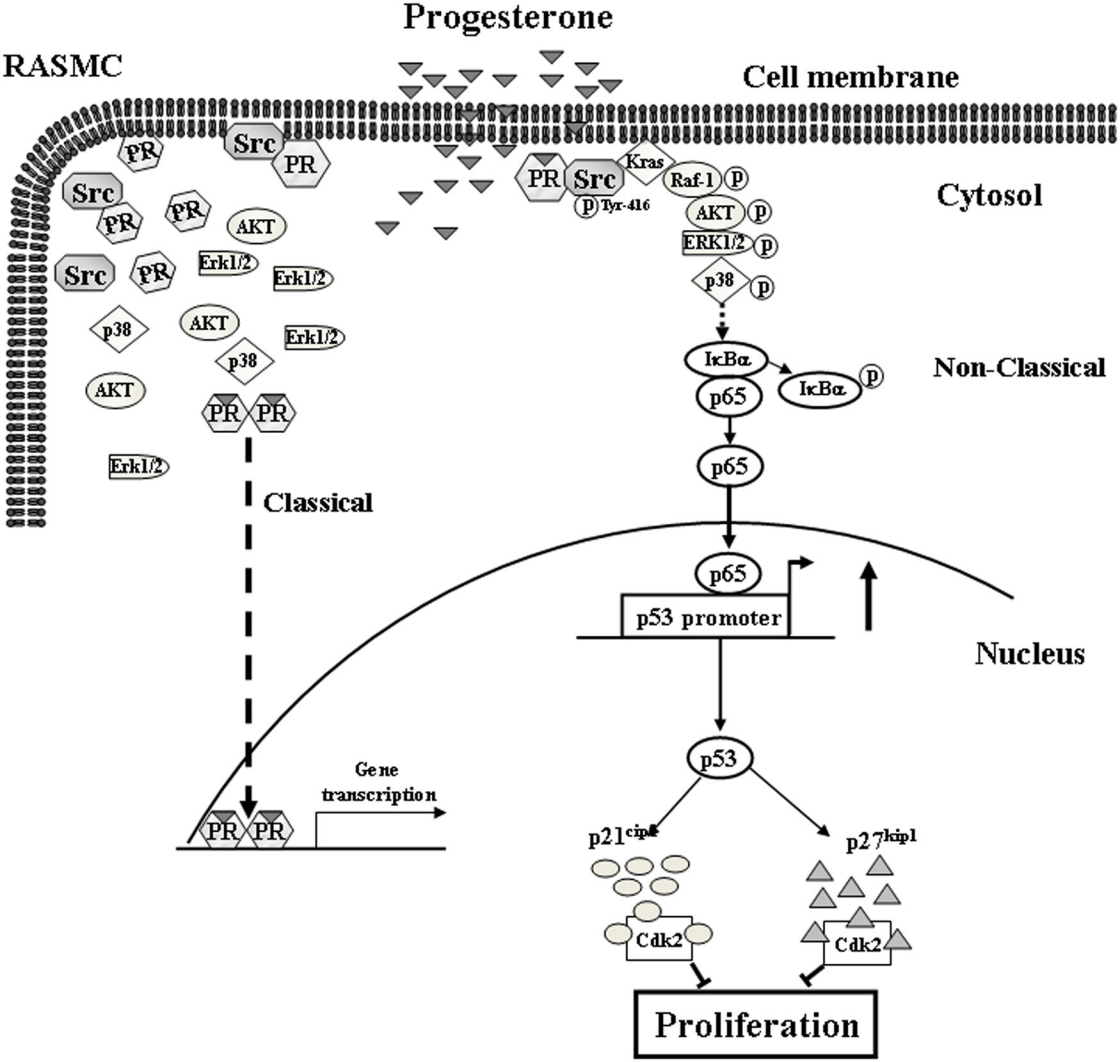
Model for P4-induced up-regulations in the levels of p21^cip1^ and p27^kip1^ protein in RASMCs. P4 activated cSrc, subsequently causing membrane translocation of Kras, which in turn activated Raf-1/AKT/ERK1/2/p38/IκBα and increased nuclear translocation of NFκB (p65), and eventually increasing the levels of p21^cip1^ and p27^kip1^ protein through up-regulating p53 expression in RASMCs. PR, progesterone receptor.

In conclusion, this study provides evidence that P4 activated the cSrc/Kras/Raf-1/AKT/ERK1/2/p38/IκB/NFκB signaling pathway, which in turn up-regulated the expression of p53, eventually up-regulating the levels of p21^cip1^ and p27^kip1^ protein in RASMCs. Based on the results from the present study and our previous study, we propose a model of the molecular mechanism underlying P4-induced cell cycle arrest in RASMCs as shown in Fig 6. The findings from the present study highlight the molecular mechanisms underlying P4-induced anti-proliferation in RASMCs.
